# Clostridioides difficile in West Africa: a systematic review of the limited evidence and key knowledge gaps

**DOI:** 10.1016/j.gloepi.2026.100281

**Published:** 2026-08-18

**Authors:** Mary-Theresa Usuanlele, Conrad Kabali, Lawrence Mbuagbaw, Mark Loeb, Dominik Mertz

**Affiliations:** a Department of Health Research Methods, Evidence, and Impact, McMaster University, 1280 Main Street West, Hamilton, Ontario L8S 4L8, Canada; b Division of Epidemiology, Dalla Lana School of Public Health, University of Toronto, 155 College Street, 6th Floor, Toronto, ON M5T 3M7, Canada; c Department of Medicine, McMaster University, 1280 Main Street West, Hamilton, Ontario L8S 4L8, Canada; d Michael G. DeGroote Institute for Infectious Disease Research, McMaster University, 1280 Main Street West, Hamilton, Ontario L8S 4L8, Canada; e Department of Anesthesia, McMaster University, 1280 Main Street West, Hamilton, Ontario L8S 4L8, Canada; f Biostatistics Unit/The Research Institute, St Joseph's Healthcare, 50 Charlton Avenue East, Hamilton, Ontario L8N 4A6, Canada; g Department of Pathology and Molecular Medicine, McMaster University, 1280 Main Street West, Hamilton, Ontario L8S 4L8, Canada

**Keywords:** West Africa, *Clostridioides difficile*, Occurrence, Knowledge gaps, Strain types

## Abstract

**Objectives:**

Despite high diarrheal burden and antibiotic use, *C. difficile* infection in West Africa is insufficiently studied. This review synthesizes the limited and methodologically diverse evidence on *C. difficile* in West Africa, summarizing study-level findings on detection, settings, risk factors and strain characteristics to clarify current knowledge gaps and priorities for future research.

**Methods:**

A systematic literature search was conducted through September 9, 2024, across major global and African databases. The methodological quality, strengths, limitations, and potential sources of bias of included studies were critically evaluated. Due to heterogeneity, findings were synthesized narratively rather than through meta-analysis.

**Results:**

Across the nine publications from eight studies identified from Nigeria, Ghana, Côte d'Ivoire, and Mali, investigators reported a wide range of findings related to *C. difficile* detection in both symptomatic and asymptomatic participants. Individual studies also described various participant or clinical characteristics examined within their samples, including demographics, co-infections, antibiotic use, nutritional indicators, and living conditions. Genotyping data, available from only a few studies, showed considerable ribotype diversity, with RT078 detected in a Malian isolate and RT084 most frequently reported in Ghana and Mali.

**Conclusions:**

The true burden of *C. difficile* in West Africa remains unknown. Existing evidence comes from a small number of heterogeneous studies conducted in only four countries, providing an insufficient foundation for regional epidemiologic understanding. Collectively, these data underscore major gaps in surveillance and diagnostic capacity; addressing them will require strengthened laboratory infrastructure, increased clinical awareness, and well-designed epidemiological studies to clarify the organism's role and inform appropriate management.

**Summary:**

This systematic review highlights the rising concern of *Clostridioides difficile* in West Africa, urging improved diagnostics, clinical awareness, and research to understand its burden and guide effective treatment.

## Introduction

*Clostridioides* (formerly *Clostridium*) *difficile* is the causative agent of *Clostridioides difficile* infection (CDI), an antibiotic-associated and often health care-associated infective diarrhea. *C. difficile* gained international recognition in the early 2000s with the report of several outbreaks in several North American and European countries [1].

The epidemiology and burden of CDI in resource-rich countries in Europe and North America are well described where it is associated with significant morbidity, mortality, and economic burdens [2], [3]. On the other hand, there is a paucity of data on CDI cases and burden in Africa, Asia and other resource-limited countries [2], [4].

Diarrheal diseases cause major morbidity and mortality in Africa [1]. They were the third leading cause of death in Africa in 2019 with about 496 deaths per 100,000 population [5]. Despite this high burden, CDI has a very low index of clinical suspicion in patients with community-acquired or hospital-acquired diarrhea [[6], [7]].

Limited diagnostic resources and surveillance practices contribute to this low clinical suspicion [8]. Perhaps with the exception of South Africa, which issued a guideline for the diagnosis, management and infection prevention and control of *C. difficile* in 2000 [6], no African country is known to conduct systematic routine surveillance for *C. difficile* [1]. Neglecting CDI in this region risks missing opportunities to reduce morbidity and mortality, promote antibiotic stewardship, and identify gaps for future research. [9].

Unregulated use of broad-spectrum antibiotics in Africa is common; this not only drives antimicrobial resistance but also increases the risk of CDI [1], [2]*. C. difficile* is a leading cause of diarrhea among HIV-infected persons [[7], [8], [10]]. With high regional diarrhea rates and about 4.8 million people living with HIV in West and Central Africa [11], *C. difficile* may contribute significantly to diarrheal illnesses in the region [4]. Additionally, the environment, animals and foods can be sources of community-acquired *C. difficile* infections in humans [[1], [12], [13], [14], [15]]; sometimes harbouring the same ribotypes found in human infections [12].

Previous reviews on CDI in Africa show limited data [1], [4]. In a literature review examining CDI among adults in sub-Saharan Africa, Keeley et al. concluded that the impact of CDI in the region remains largely poorly understood. They however found a good description of toxigenic *C. difficile* carriage, which was often associated with antibiotic usage in most of the studies found [4]. And in a narrative review investigating the incidence, prevalence, molecular epidemiology and antimicrobial susceptibility of *C. difficile* strains in Africa, Kullin et al. found that CDI may be a significant, under-reported cause of diarrhea across much of Africa and emphasized that while active surveillance is difficult, small, well-designed studies are key to understanding its true burden [1].

*C. difficile* colonization refers to the presence of the organism (toxigenic or non-toxigenic) without clinical symptoms, whereas *C. difficile* infection (CDI) requires both symptoms (typically diarrhea) and evidence of toxigenic *C. difficile*. This distinction is important for interpreting epidemiologic data.

Keeley et al. focused solely on adult CDI cases in sub-Saharan Africa, while Kullin et al. limited their review to studies published between January 1995 and June 2021. In contrast, our review examined studies on *C. difficile* in West Africa (WA) without restrictions on age or publication date.

This systematic review aimed to synthesize available evidence on *C. difficile* carriage in WA, across hospital and community settings. It summarizes reported detection and strain-typing findings. The review highlights the fragmented nature of existing data and outlines the major gaps that constrain understanding of the organism's role in the region.

## Methods

We conducted this review using established methodologies and reported it in accordance with the Preferred Reporting Items for Systematic Reviews and Meta-Analyses (PRISMA) 2009 checklist [16]. The review protocol was prospectively registered in the International Prospective Register of Systematic Reviews (PROSPERO; registration number CRD42024603011). The eligibility criteria remained consistent with the registered protocol throughout the review process. However, following peer review and editorial feedback, several methodological aspects, including the presentation and synthesis of findings, were revised from the original protocol. Specifically, the review evolved from a prevalence-focused synthesis to a narrative synthesis of study-level detection findings and evidence gaps. These modifications did not alter the overall objectives of the review or the study selection criteria and are reflected in the methods reported herein.

### Search strategy

A systematic literature search from database inception up to September 9, 2024, was performed across the following databases: MEDLINE (PubMed), Embase, CENTRAL, Web of Science, Scopus, the World Health Organization Global Health Libraries, regional database (African Index Medicus), and African Journals Online. Search terms included “*Clostridium difficile*,” “*Clostridioides difficile*,” “*C. difficile*,” “CDI,” “antibiotic-associated colitis,” “pseudomembranous colitis,” and “pseudomembranous enterocolitis.” The strategy focused exclusively on West African countries using MeSH terms, Title/Abstract keywords, and the specific names of the countries. Reference lists from evidence syntheses including narrative reviews, systematic reviews, meta-analyses, and guidelines were screened to identify additional relevant articles. A PhD dissertation was also encountered during an unrelated Google Scholar search. As it met the review's inclusion criteria, it was evaluated and incorporated into the study.

### Study selection and analysis

All interventional and observational studies reporting the prevalence and/or incidence of *C. difficile* in humans in WA were eligible for inclusion, regardless of language, date, or publication status. Studies involving adult or pediatric CDI cases, patients with or without diarrhea or other symptoms, tested for *C. difficile* by bacterial culture, enzyme immunoassay (EIA) for Glutamate Dehydrogenase (GDH)/toxin A/B, Nucleic Acid Amplification Test (NAAT), or colonoscopy/sigmoidoscopy for pseudomembranous colitis were eligible. NAAT is widely accepted as a sensitive and specific method for detecting toxigenic *C. difficile*, and international guidelines [17] consider NAAT an appropriate component of multi-step diagnostic algorithms. Given the heterogeneity of diagnostic capacity across WA, including NAAT-based studies allowed us to capture all available evidence on toxigenic *C. difficile* while transparently acknowledging differences in diagnostic approaches. Global studies with extractable data specific to WA were also considered. Eligible study designs included randomized and non-randomized trials, cohort and ecological studies, as well as conference abstracts, unpublished presentations, economic analyses, and grey literature. We excluded animal studies, opinions, correspondences, and letters to the editor.

Identified studies were uploaded to Covidence, and duplicates removed. Two reviewers (MU and CK) independently screened titles and abstracts using the eligibility criteria described above. Articles deemed eligible by either reviewer were included. Full-text screening was then conducted independently, and any disagreements were resolved through discussion.

Data were independently extracted by MU and CK using a standardized form. All disagreements were resolved through discussion. Extracted details included study citation, design, publication year, country, duration, participant demographics (age, gender, comorbidities), sample sizes, reported findings on *C. difficile* detection, and whether cases occurred in community or hospitalized settings.

Two reviewers, MU and CK, independently conducted a critical appraisal of the included studies, examining methodological rigor, strengths, limitations, potential sources of bias, and the overall reliability of the evidence. Any discrepancies in assessment were resolved through discussion and consensus.

A proportional meta-analysis stratified by country, testing method, and age group was initially planned to estimate the prevalence or incidence of *C. difficile*. However, considerable heterogeneity in study methodologies, populations and diagnostic techniques limited statistical pooling. Consequently, a narrative synthesis of findings was undertaken. Meta-Analysis Shiny App was used to present the point estimates of each included study without generating a pooled estimate [18].

## Results

**Fig. 1:**
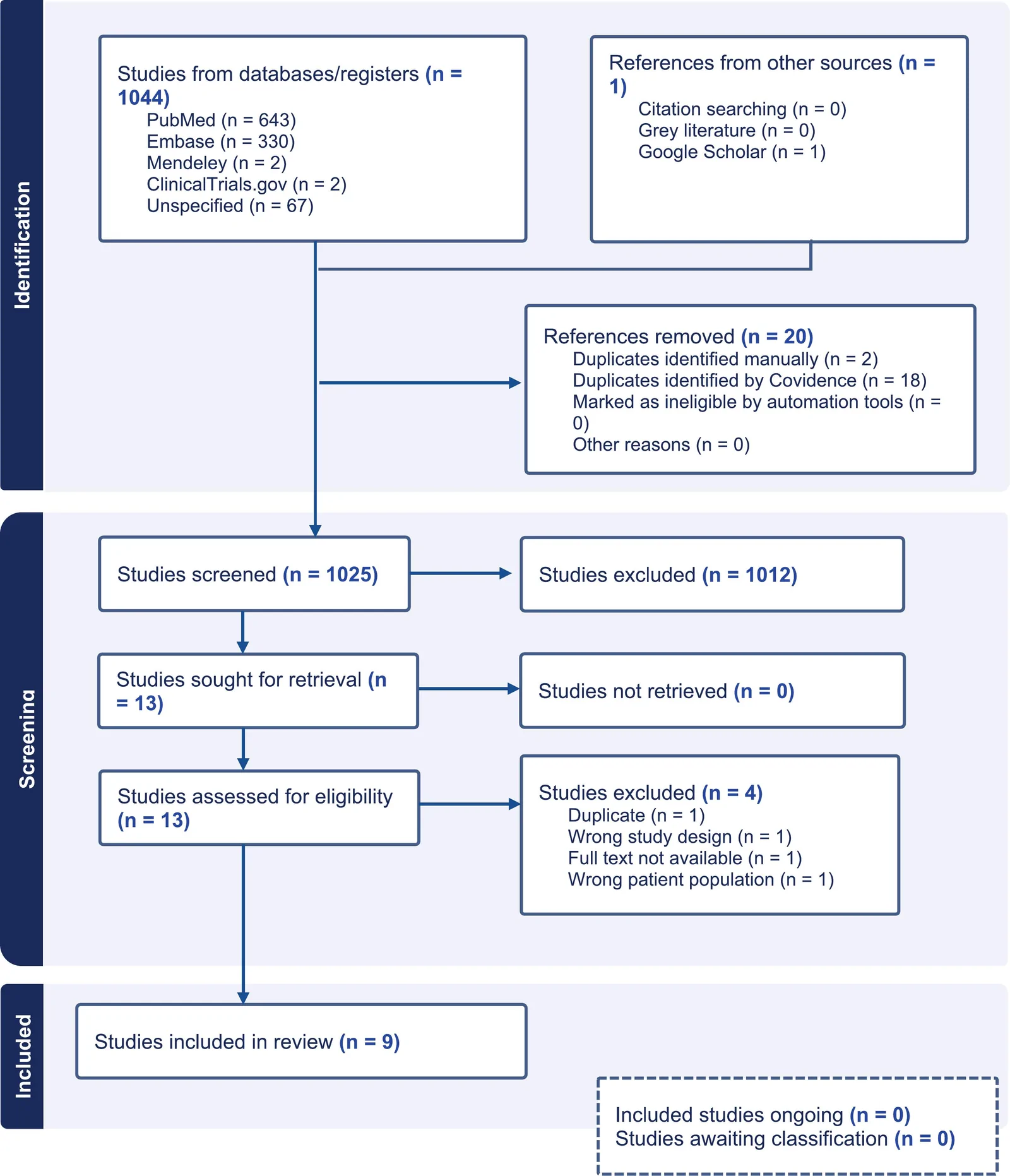
Prisma flow diagram. *Clostridioides difficile* in West Africa: A systematic Review of limited evidence and key knowledge gaps.

The search yielded 1045 citations; after removing 20 duplicates, 1013/1025 (98.8%) were excluded by reviewing titles/abstract. Of the remaining 12 articles, 8 (67%) were included after full text review. Two studies reported on the same dataset [[19], [20]]. One relevant dissertation from Mali [21], identified via Google Scholar was added for a total of nine publications representing eight distinct studies (Fig. 1).

The included studies were published between 1986 and 2023 and comprised 6 from Nigeria [[19], [20], [22], [23], [24], [25]], and one each from Cote d'Ivoire [26], Ghana [27] and Mali [21]. A detailed study-level characteristics and findings are summarized in Table 1.

**Table 1:** Characteristics of Included Studies.

Author/Year/ Ref Study Design	Country	Setting Aims of study	Outcome Colonization vs Infection	Sample size Study population	Diagnostic methods	Results	Ribotypes	Associated contextual factors.
1. Becker et al., 2015 [26] Case control	Cote d'Ivoire	Community **AIM:** To report on the frequency and characterization of *C. difficile* strains.	Not defined	Total = 298 Cases = 68 Controls = 230 Individuals aged ≥1 year. **Cases:** persistent diarrhea ≥2 weeks. **Controls:** No gastrointestinal symptoms	RDT - Clostridium K-SeT, Coris BioConcept, Gembloux, Belgium to detect GDH. Anaerobic culture for *C. difficile* detection and multiplex PCR (GenoType CDiff, Hain Lifescience, Nehren, Germany) to detect GDH*, tpi, tcdA*, *tcdB* and *cdtA/B.*	16 GDH-positives: 4 (6%) of cases and 12 (5%) of controls. Toxigenic = 0 Non-toxigenic = 6 (2 cases vs 4 controls (*p* = 0.6)	RT199 = 1 RT390 = 1 New RTs = 4	Parasitic coinfections
2. Janssen et al. 2016 [27]. Prospective cross-sectional	Ghana	Hospital and community. **AIMS:** Determine the prevalence of *C. difficile* and explore risk factors for colonization. Investigate association between HIV and *C. difficile* colonization.	Not defined	Total = 307 Diarrhea = 176 No diarrhea = 131 Individuals of all ages. Hospitalized patients with diarrhea vs volunteers from community with no diarrhea	EIA (C. DIFF QUIK CHEK COMPLETE (®), Techlab, Blacksburg, USA) for simultaneous detection of toxin A + B + GDH. Identification of *C. difficile* was confirmed by recultivation and MALDI-TOF mass spectrometry using Biotyper (Bruker Daltonics, Bremen, Germany)	15*C. diff* positives 8 (5%) diarrheal, 7 (5%) non-diarrheal. Toxigenic = 3 CDT = 1	RT 084 (*n* = 6). RT 011/049 (*n* = 1) RT 097 (n = 1) RT SLO 091 (n = 1) RT SLO 095 (n = 1) RT SLO 228 (n = 1) RT SLO 233 (*n* = 2) RT SLO 235 (n = 1) RT SLO 237 (n = 1)	Parasitic coinfections Higher proportions of *C. difficile* were seen in children <5 years (*p* = 0.004), those receiving ceftriaxone (*p* = 0.02), and individuals with malaria (*p* = 0.04) or rashes (*p* < 0.001).
3. Oguike and Emeruwa, 1990 [20] AND 4. Emeruwa and Oguike, 1990 [19] (Two publications from one study) Cross-sectional	Nigeria	Outpatients **AIMS:** Investigate frequency of *C. difficile* and colonization by toxin-producing strains and the impact of modes of infant feeding,	Not defined	Total = 320 Diarrhea = 61 No diarrhea = 259 Children under 5 years of age visiting hospitals and health centers.	Culture media and tissue culture for cytotoxin production.	156*C. difficile*-positive cultures: 47 diarrheal, 109 non-diarrheal; cytotoxin-positive in 23 (7 diarrheal, 16 non-diarrheal).	Not investigated .	*C. difficile* occurred more in Rural dwellers 1 day to 1 year olds Mixed-fed children
5. Adesoji et al., 2023 [23] Cross-sectional	Nigeria	Outpatients **AIMS** Estimate the occurrence of *C. difficile* among pediatric diarrheic outpatients.	Not defined	Total = 76 Pediatric patients ≤2 years old to 17 years old presenting with diarrheal	Toxigenic culture to detect *C. difficile* followed by PCR of the isolates. Cdifftox activity assay to detect toxin activity and *C. difficile* TOX A/B II ELISA test (TechLab, Blacksburg, VA, USA) to detect toxin production.	Culture positive = 31 Toxigenic = 31	Not investigated	More *C. difficile* with recent antibiotics (*p* = 0.03) and awareness (*p* = 0.05); higher in urban vs rural females (*p* = 0.5 vs 0.2) and males (*p* = 0.6 vs 0.2).
6. Onwueme et al., 2011 [22] Cross-sectional	Nigeria	Outpatients and In-patients. **AIMS** Estimate toxinogenic *C. diff* prevalence among persons with HIV	Not defined	Total = 140 97 HIV+ 43 HIV- Adults patients with diarrhea.	Inverness Medical Professional Diagnostics, Princeton, NJ, USA, a rapid enzyme immunoassay (EIA) which detects *C. difficile* toxin A or B.	*C. difficile* toxin detected in 21 diarrheal samples.	Not investigated	*C. difficile* more prevalent in outpatients living with HIV (*p* = 0.007)
7. Rotimi and Akindutire, 1989 [25] Case-control	Nigeria	Out-patients and In-patients. **AIM** Investigate *C. difficile* incidence in diarrheal patients with antibiotic exposure and in healthy adult controls.	Not defined	Total = 79 Diarrhea = 41 No diarrhea = 38 Adult patients and healthy volunteers	Stool culture for *C. difficile* on selective media. Cytotoxin production by cell cytotoxicity neutralization assay (CCNA).	35*C. diff* culture positive. Diarrhea = 23/41 Non-diarrhea = 12/38 Toxigenic = 24 Cytotoxin positive in stool = 1	Not investigated	Prior antibiotic exposure
8. Rotimi and Akindutire, 1986 [24] Cross-sectional	Nigeria	Patients and volunteers **AIM** Investigate the occurrence of *C. difficile* in non-diarrheal stool specimens for baseline data to inform it's role in adult African diarrheal diseases.	Not defined	**Part 1:** 324 non-diarrheal adult fecal specimens. **Part 2:** 36 healthy adults sampled pre-antibiotic (Day 1), during therapy (Days 1–5), and post-therapy (Days 5, 10, 20, 3 mo).	Stool culture on selective media for *C. difficile.*	**Part 1**: 177/324 (55%) *C. difficile*-positive. **Part 2**: Pre-antibiotic: 12/36 (33%); During therapy Days 1–5: 36/36 (100%); Post-therapy: Day 5 = 20 (56%), Day 10 = 15 (42%), Day 20 = 14 (39%), 3 mo = same as Day 20.	Not investigated	*C. difficile* was more prevalent in females than in males (*p* < 0.05). Antibiotic exposure
9. Schwarzkopf, 2023 [21]. Case-control	Mali	Hospital and Community **AIM**: Investigate presence of *C. difficile* in southern Mali in patients with diarrhea and asymptomatic controls.	Not defined	Total = 233; Bamako: 180 (83 cases, 97 controls); Nonio: 53 (cases/controls unspecified). Individuals aged 10 days to 75 years old	TECHLAB (®) C.DIFF QUICK CHEK COMPLETE kit from Alere Diagnostics, a rapid membrane enzyme immunoassay which detects both GDH and toxins A and B followed by multiplex PCR & culture.	13 (7%) Bamako specimens were GDH positive 12 (7%) were culture and PCR positive. 1 toxin A + B+ and CDT positive.	RT 084 (*n* = 7). RT 078 (*n* = 1) Unknown RTs (*n* = 4)	*C. difficile* more prevalent with presence of diarrhea (*p* = 0.007)

Footnote:

RDT – Rapid Diagnostic Test.

GDH - Glutamate Dehydrogenase.

PCR - Polymerase Chain Reaction.

*Tpi -* Triose phosphate isomerase.

*tcdA -* The gene for Toxin A.

*tcdB -* The gene for Toxin A.

RT – Ribotype.

HIV - Human Immunodeficiency Virus.

EIA - Enzyme Immunoassay.

SLO – Slovenian *C. difficile* strains.

ELISA - Enzyme-Linked Immunosorbent Assay.

CCNA - Cell Cytotoxicity Neutralization Assay.

Study designs included three case-control [[21], [25], [26]] and five (six publications) cross-sectional studies [[19], [20], [22], [23], [24], [27]]. Participants included adults only [[22], [24], [25]], pediatrics only [[20], [23]], and mixed-age groups [[21], [26], [27]] across varied settings - community volunteers, outpatients, inpatients, and both rural and urban populations. Studies conducted before 2000 [[19], [20], [24], [25]] typically used stool cultures and cell culture cytotoxicity neutralization assay (CCCNA) to detect *C. difficile* and its toxins. Post-2000 studies [[22], [23], [26], [27]] shifted toward immunogenic and molecular methods.

Critical appraisal of the included studies identified several methodological limitations that may affect the strength and generalizability of the findings. Most studies used non-random sampling methods, primarily convenience sampling [[20], [21], [23], [25], [26], [27]], while one used purposive sampling [22] and one used consecutive sampling [24], thereby introducing potential selection and coverage biases.

### Reported findings on *C. difficile* detection

All nine studies with a total of 1813 participants reported findings on *C. difficile* detection. Across these individual studies, the number of participants testing positive varied widely, from 5% (12/230) [26] in asymptomatic individuals to 77% (47/61) in symptomatic children under five [20]. No study reported incidence data. Fig. 2 (supplementary material) shows the forest plot of study level findings and Fig. 3 (supplementary material) shows the forest plot of proportion of *C. difficile* colonization among symptomatic and asymptomatic individuals.

We identified three studies with four publications [[19], [20], [23], [27]] that reported on pediatric participants. In a cross-sectional study in Eastern Nigeria involving 320 children under the age of five, *C. difficile* was isolated from 156 stool samples (49%). Among these, 15% (23/156) were identified as toxigenic strains [[19], [20]]. A second study among 76 pediatric outpatients visiting three hospitals in Northern Nigeria, identified *C. difficile* in 31 samples (41%), all of which were positive for toxins A and B [23]. Finally, among 86 pediatric participants in a study from Ghana, 8/86 (9%) tested positive for *C. difficile* in the subgroup of pediatric participants [27].

Another three studies [[22], [24], [25]] focused on adult participants. Onwueme et al. found toxigenic *C. difficile* in 22% (21/97) of HIV-positive symptomatic patients in a cross-sectional study at two urban Nigerian hospitals [22]. Another study from Nigeria, a case-control study of 41 antibiotic-exposed adults with diarrhea and 38 healthy controls found 35 of 79 (44%) *C. difficile*-positive individuals with toxigenic strains in 18/35 (51%) [25]. A previous study by the same authors among 324 healthy adults (15–62 years) in Nigeria identified 55% (177/324) of specimens as positive. Finally, in the subgroup of adult patients in the study from Ghana, 7/221 (3%) patients tested positive [27].

Three studies [[21], [26], [27]] included mixed populations where pediatric and adult patients could not be separated. In a case-control study in Côte d'Ivoire, 5% (16/298) of stool specimens were GDH positive, six (38%) of which were non-toxigenic strains [26]. In another study in rural Western Ghana, 15/307 (5%) were GDH-positive, 3 (20%) of which were toxin-producing strains. Finally, in a case-control study in Bamako, Mali comparing 83 participants (with diarrhea) to 97 (without diarrhea), a total of 12/180 (7%) tested positive for *C. difficile*. Depending on the test used, one (8%) or three (25%) were reported as toxin-producing strains [21]. Fig. 4 (supplemental material) is a forest plot showing the proportion of *C. difficile* colonization in adult, pediatric and mixed populations.

Ribotyping was performed in three studies [[21], [26], [27]] on a total of 33 isolates. The following ribotypes (RT) were identified in Côte d'Ivoire study: RT 199 (*n* = 1), RT 390 (*n* = 1), and four novel ribotypes among the six non-toxigenic *C. difficile* isolates [26]. In Ghana, the 15*C. difficile* isolates included RT 084 (*n* = 6) as most common, along with one each of RT 011/049, RT 097, RT SLO 091, RT SLO 095, RT SLO 228, RT SLO 233, RT SLO 235 and RT SLO 237 [27]. In Mali, the 12 culture-positive isolates included RT 084 (*n* = 7), RT 078 (*n* = 1), and four unclassified RTs isolates [21].

### Contextual factors examined in included studies

Individual studies examined a range of participant and contextual characteristics in relation to *C. difficile* detection; these included the presence of diarrhea, age, sex, presence of other pathogens and parasites, antibiotic exposure, nutrition and residential settings (urban vs rural).

#### Presence of diarrhea

For the purposes of this review, diarrhea was considered a reported clinical feature rather than an indicator of colonization or infection, as the included studies generally did not differentiate these outcomes or establish *C. difficile* as the cause of symptoms.

Five included studies, three case-control [[21], [25], [26]] and two cross-sectional studies in three publications [[19], [20], [27]], reported *C. difficile* detection in both symptomatic and asymptomatic individuals.

In one study conducted in Dabou and surrounding villages in Côte d'Ivoire, non-toxigenic *C. difficile* was found in similar proportions among symptomatic participants (6%, 4/68) and asymptomatic controls (5%, 12/230) [26]. In Bamako, Mali, *C. difficile* was found in 2/83 (2%) symptomatic cases vs 10/97 (10%) healthy controls. Only one isolate was toxigenic [21]. In one Nigerian study, *C. difficile* was cultured in 56% (23/41) of diarrheal cases and in 32% (12/38) of healthy controls with toxigenic strains present in similar proportions in both groups (70%, 16/23) and (67%, 8/12) respectively [25].

In Eikwe, Ghana, *C. difficile* was found in 5% (8/176) of symptomatic and 5% (7/131) of asymptomatic participants. Three toxigenic strains (TcdA/B) were detected; two from symptomatic vs one from healthy participants [27].

In a study conducted in Eastern Nigeria, *C. difficile* was detected in 77% (47/61) of diarrheal vs 42% (109/259) of non-diarrheal children under the age of five, respectively. The proportion of toxigenic strains was similar for both diarrheal patients and non-diarrheal patients, i.e. 15% (7/47) vs 15% (16/109) respectively [[19], [20]].

#### Age and sex

In one Ghanian study, children under the age of five had higher observed rates of *C. difficile* colonization compared to older participants (53%; 8/15 vs 46%; 7/15) [27]. Similarly, in the eastern Nigerian study, the highest rates were found in infants (58% in 0–1-year-olds and 50% in 1–2 years olds) to then decline sharply with age (32% in 2–3-year-olds, 11% in 3–4 year olds and 6% in 4–5 year olds). Cytotoxin positivity was also highest in infants: 17% (20/120) among 0–1-year-olds, 10% (2/20) among 1–2 years olds and 8% (1/12) among 2–3-year-olds. No cytotoxin producing isolate was found in children >3 yrs. [[19], [20]]. In another Nigerian pediatric outpatient study, the highest rates were found in 0–2 years old (53%) and lower rates in older age groups (40% among 2–12 year olds and 7% among 13–17 year olds) in northern Nigerian pediatric outpatients [23]. The same study reported differing proportions by sex, with higher detection among males than females, (65% vs 35%) [23]. In contrast, a study among healthy adults in Nigeria, found higher rates in females (90/146; 62%) than males (87/176; 48%) [24].

#### Intestinal pathogens and parasites

Becker et al. found one co-infection (*Blastocystis* spp., *Entamoeba coli*, and *E. hartmanni)* among six positive patients, while 5/9 samples showed co-infections with various protozoans in *C. difficile* negative patients (*Endolimax nana*, *Giardia intestinalis*, *E. coli*) and *a helminth (*hookworm) [26].

In the Ghanaian study, malaria was more frequently observed among participants who tested positive for *C. difficile* (20%; 3/15) vs those who tested negative (5%; 13/274) [27].

#### Antibiotics

Janssen et al. (2016) reported that current ceftriaxone use was more frequently observed among participants who tested positive for *C. difficile* (2/15; 14%) which was not the case with prior use. Adesoji et al. (2023) observed higher proportions of pediatric *C. difficile* detection among children with recent antibiotic use within the last 14 days (11/18; 61% vs 14/44; 32%) among those without recent antibiotic use. They also found higher proportions among those with greater knowledge about antibiotics (14/25; 56%) than among those without antibiotic knowledge (16/49; 33%) [23]. In a small prospective cohort sub-study, the proportion of participants with positive *C. difficile* cultures increased from 12/36 (33%) prior to clindamycin exposure to 100% during the 5-day exposure period, then declined to 56%, 42%, and 39% on days 5, 10, and 20 post-treatment. The 12 participants who were positive at baseline remained positive at 3 months. The study did not distinguish incident from prevalent colonization [24]. In contrast, Schwarzkopf (2023) did not find meaningful differences between recent antibiotics use and *C. difficile* detection in Mali. None of the participants who tested positive for *C. difficile* reported recent antibiotic use [21].

#### Nutrition

One study of 320 children under five reported *C. difficile* detection in 42% of exclusively breast-fed infants, in 33% of exclusively formula-fed infants and in 66% of mixed-fed infants. Cytotoxin-positive isolates were recovered in 18% of breast-fed, 50% of formula-fed, and 19% of mixed-fed children [[19], [20]]. The Malian study [21], which included a mixed-age population, and reported on general nutritional characteristics did not find any correlation between *C. difficile* colonization and eating habits.

#### Socio-cultural factors

In one study, nearly half of the participants (46%) reported regular contact with household animals including sheep, cattle, and poultry. However, the proportion of *C. difficile*–positive participants who reported contact with household animals (5/13; 39%) did not differ meaningfully from those without such exposure (8/13; 62%). This difference maybe due to chance alone. Despite varied drinking water sources (88% of respondents consumed purified water (tap or bottled), 9% drank spring water and 2% drank untreated river water), *C. difficile* colonization did not vary appreciably across these groups. There was also no clear pattern with household crowding [21].

#### Urban vs rural residency

In the Eastern Nigerian study, the numbers of children under five who tested positive for *C. difficile* differed between rural and urban settings (53% vs 48% in Nsukka and 48% vs 42% in Enugu) although cytotoxin-positive proportions were similar across settings [[19], [20]]. A later study from Northern Nigeria reported the opposite pattern with higher detection among urban pediatric (2–12 years) outpatients [23]. In another study from Mali, all positive cases (*n* = 12) were found to come exclusively from urban residents, no rural resident tested positive for *C. difficile* [21].

#### Other contextual factors

This same study in Mali did not find any meaningful differences between *C. difficile* detection and factors such as current hospitalization, prior hospitalization, regular medication, and weight loss [21].

In one study in Ghana, rashes were more common among participants who tested positive for *C. difficile* (6/15; 40%) [27]. The same study found no differences by HIV status; all participants living with HIV testing negative [27]. In contrast, a Nigerian study reported high proportions of toxigenic *C. difficile* (11/74; 15%) among diarrheal outpatients living with HIV compared with HIV-negative outpatients, in whom no toxigenic isolates were detected (0/43; 0%) [22].

## Discussion

This systematic review describes the available evidence on *C. difficile* in West Africa, summarizes study-level findings on circulating strains, and identifies key evidence gaps. Nine studies from four countries in West Africa —Nigeria, Ghana, Côte d'Ivoire, and Mali—were identified. This included 1813 participants of all ages across all included studies, highlighting gaps in regional data and the need for broader surveillance across the continent.

CDI is not routinely tracked across WA, where health systems prioritize high-burden diseases like malaria, TB and HIV [[28], [29]], leading to inconsistent testing and underreporting. Some Nigerian studies reported apparently higher frequencies of *C. difficile* carriage and toxigenic strain detection (up to 55% among asymptomatic adults) than those reported in some global studies (2–17% among healthy adults). However, such comparisons should be interpreted cautiously because of differences in study populations, sampling approaches, and laboratory methodologies [[30], [31], [32], [33]]. The apparently higher occurrence observed in the Nigerian studies may be attributable to methodological factors, such as culture-based diagnostic methods with enrichment, hospital-based sampling, and small study populations, rather than true underlying differences in occurrence. Culture-based methods with enrichment, as used in the Nigerian studies, are known to increase detection of asymptomatic colonization [[17], [34]]. The Nigerian studies sampled hospitalized adults, a population known to have higher colonization rates due to recent antibiotic exposure and healthcare contact [[32], [33], [35]]. The small sample sizes and absence of community controls in the Nigerian studies limit comparability with global prevalence estimates [[17], [32]].

Studies that assessed co-infections did not report a consistent pattern of co-occurrence between *C. difficile* and other intestinal pathogens. Parasitic infections were reported sporadically, and no clear relationship with symptom status was described. [[21], [26], [27]]. Co-infections with intestinal parasites may affect *C. difficile* severity [36], complicating diagnosis and treatment. Clinicians should consider them when evaluating diarrhea cases in similar settings [37]. One Ghanaian study, reported higher proportions of malaria among participants who tested positive for *C. difficile* [27]. However, this observation was based on a single study, and no consistent pattern of co-occurrence was identified across the included evidence. While malaria may co-occur with bacterial infections, such links to *C. difficile* remain unestablished [38].

A Nigerian study reported high proportions of toxigenic *C. difficile* among outpatients living with HIV [22], whereas similar findings were not reported in studies from Ghana or Mali [[27], [39]]. Although studies from the United States have described *C. difficile* as a cause of diarrheal illness among people living with HIV [10] especially in those with low CD4 counts <50 cells/mm^3^ [[36], [37]]. However, the limited and heterogeneous evidence from West Africa precludes conclusions regarding the occurrence or clinical significance of *C. difficile* infection in this population. Clinicians should consider CDI in HIV-related diarrhea cases [40].

Among children, *C. difficile* colonization declines with age [[41], [42], [43], [44]]. Studies from Ghana and Mali reported higher frequencies of *C. difficile* detection among younger children, particularly those under five years of age in Ghana [27] and in infants under one in Mali [21] compared to adult cases. Although a Nigerian study showed unexpectedly higher rates in young adults [25], across the Nigerian studies, the findings were generally consistent with the well-established age-related decline with increasing age [[19], [20], [23]]. Overall, the included studies suggest variation in *C. difficile* detection across age groups, although differences in study populations, sampling approaches, and diagnostic methods limit direct comparisons.

*C. difficile* rates are often higher in females than in males [[45], [46], [47]] as observed in a Nigerian and the Malian studies [[21], [24]]. However, one Nigerian pediatric study found the opposite with higher rates in males (65%) than in females (35%) [23]. These findings were not consistent across studies, and the limited evidence does not permit conclusions regarding sex-related patterns of *C. difficile* occurrence in West Africa. A higher incidence of *C. difficile* in females may reflect their greater healthcare use and antibiotic exposure, potentially increasing susceptibility to infection [[48], [49]].

Antibiotics, especially broad-spectrum ones, disrupt gut flora, promoting *C. difficile* colonization [50]. Higher proportions of *C. difficile* was reported among participants with recent antibiotic exposure in a Ghanaian and a Nigerian study [[23], [27]]. A 100% infection or colonization rate was observed during treatment with clindamycin as compared to 33% pre-treatment [24]. In the Malian study, no clear pattern was found with prior antibiotic use, potentially explained by limitations in the methods such as self-reporting of antibiotic exposure which may have been unreliable [21]*.*

Infant feeding mode may influence *C. difficile* colonization. In a Nigerian study, *C. difficile* occurred more among mix-fed infants (66%) than breastfed infants (42%) or formula-fed infants (33%) [[19], [20]], contrasting earlier studies [[51], [52]] and data from Alberta which showed higher occurrence in formula-fed infants [53]. Given the limited evidence available and the inconsistency of findings across settings, no conclusions can be drawn regarding the relationship between infant feeding mode and *C. difficile* occurrence in this review.

Breast milk offers passive immunity and fosters a healthier gut microbiome, with more *Bifidobacterium* spp. and fewer *Clostridium* spp. compared to formula feeding [54].

Mixed feeding may carry higher risk than exclusive breastfeeding because partial breastfeeding reduces the immunologic protection provided by breast milk. Breast milk supplies antibodies and bioactive factors that protect against enteric and respiratory pathogens, and these protective effects are strongest with exclusive breastfeeding. Introducing formula lowers infants' exposure to these protective components while leaving them vulnerable to environmental pathogens [55].

In many low-resource settings, including parts of Nigeria, formula preparation also poses hygiene challenges such as unsafe water, inadequate sterilization and poor storage practices. Thus, mixed feeding may represent a “double risk”: reduced immunologic protection and increased exposure to pathogens introduced during formula preparation [55]. These mechanisms may explain the higher occurrence among mixed-fed children in this study.

No association was found between eating habits and *C. difficile* in the Malian study [21]. The study also did not identify any clear patterns between socio-cultural associations and *C. difficile* detection [21]. In that study, the proportions of *C. difficile*–positive participants did not vary meaningfully by contact with household animals, water source, or household size, despite evidence that pets can carry *C. difficile* [56], spores can survive in tap water [[57], [58]] and crowded homes may drive community-acquired infections [59].

*C. difficile* detection showed mixed urban–rural trends. Frequency of detection was higher in rural-dwelling children in the study from Eastern Nigeria [20], but the opposite was the case in the studies from Northern Nigeria [23] and Mali [21]. Community-acquired CDI was higher in rural Northern Ireland [60], while urban and rural Ontario showed similar rates [61]. Given the limited and heterogeneous evidence, no clear pattern of *C. difficile* occurrence according to place of residence was identified in this review. Urban risks stem from dense populations and greater access to healthcare and antibiotics while livestock contact and substandard living conditions may drive rural risks [62].

Genotyping was performed in three studies [[21], [26], [27]] identifying diverse ribotypes. Only one hypervirulent strain (RT078) was found in Mali [21], however, the clinical status of the individual and disease severity were not reported. The non-hypervirulent RT084 was most common in Ghana and Mali [[21], [27]]. Other reported ribotypes were either unknown or uncommon. No Nigerian study conducted ribotyping.

Limitations of this review include potential publication bias due to study heterogeneity across included studies and lack of distinction between colonization and infection in the published literature, leaving uncertainty around the role of *C. difficile* in causing diarrhea. In addition, none of the included studies provided clinical severity data, which limits the ability to identify or evaluate potential factors associated with severe disease. No study reported CDI-associated mortality, preventing any assessment of disease outcomes in the region.

The included studies span over more than three decades, during which antibiotic access and diagnostic capacity in WA have changed substantially. Access to non-prescription antibiotics increased compared to earlier decades and remains widespread [[63], [64]]. Regional analyses also show high consumption of broad-spectrum antibiotics and rising antimicrobial pressure [65]. Diagnostic systems have similarly evolved, with recent initiatives expanding laboratory networks and decentralizing testing [66]. Current surveys indicate greater availability of bacteriology and susceptibility testing, though variability persists [67]. Although diagnostic capacity has improved in recent years, the highest *C. difficile* detection rates were reported in the oldest Nigerian studies. These early investigations relied on small, hospital-based convenience samples enriched for diarrheal or recently antibiotic-exposed patients, groups known to have higher colonization rates. Such sampling strategies likely inflated apparent detection rates despite the use of less sensitive diagnostic methods. In contrast, more recent studies have drawn from broader and sometimes community-based populations, which may partly explain their lower estimates even with improved diagnostics. Taken together, these patterns are consistent with the possibility that earlier estimates were influenced by study design and sampling approaches rather than true temporal changes in *C. difficile* carriage. Accordingly, these early estimates are best interpreted as illustrating the historical absence of surveillance and the persisting gaps in CDI epidemiologic data.

A key limitation in some of the included studies is the lack of clear differentiation between colonization and true infection. Misinterpreting asymptomatic carriage as disease results in an overestimation of the burden, especially in children which have high colonization rates. Future work should use standardized definitions and include clinical correlates to accurately differentiate colonization from infection.

The included studies exhibited methodological limitations, particularly the use of non-random sampling approaches, which constrain the strength of the evidence and limit confidence in the conclusions drawn. Rather than providing definitive estimates of *C. difficile* burden, this review underscores the limited and heterogeneous nature of the existing evidence on *C. difficile* in WA. Although our initial objectives included estimating prevalence and examining risk factors, the available data do not support robust quantitative synthesis. The heterogeneity of study designs, diagnostic methods, and reporting practices limit comparability and constrains interpretation. These limitations are themselves an important finding, highlighting substantial gaps in surveillance, diagnostic capacity, and molecular characterization in the region.

## Conclusion

The available evidence on *C. difficile* in WA remains extremely limited, with published data identified from only 4 of the 16 countries in the region. As a result, the current evidence cannot determine the regional burden of CDI, estimate prevalence, or identify risk factors with confidence. The findings instead highlight what the existing studies report at the individual study level and, more importantly, what remains unknown.

Across the included studies, *C. difficile* was detected in several clinical contexts, but the small number of investigations, inconsistent diagnostic approaches, and substantial methodological heterogeneity prevent meaningful inference about epidemiologic patterns or associations with co-infections. Observed co-occurrence with intestinal parasites or other pathogens in some studies cannot be interpreted as evidence of interaction or risk, but these findings do underscore the diagnostic complexity clinicians may face in settings where multiple infectious causes of diarrhea are common.

What this review does reveal is a substantial evidence gap. To define the true burden and epidemiology of CDI in WA, the next steps require concrete, coordinated research efforts:

• Prospective, adequately powered studies using standardized case definitions and diagnostic methods.
• Routine incorporation of toxin testing and molecular strain typing to characterize circulating lineages.
• Integration of antimicrobial stewardship data to examine contextual drivers of CDI.
• Surveillance systems capable of capturing both community- and hospital-associated disease and
• Capacity strengthening to ensure reliable laboratory detection across the region.

Until such studies are undertaken, the epidemiology of *C. difficile* in West Africa will remain poorly defined.

## Declaration of generative AI and AI-assisted technologies in the manuscript preparation process

During the preparation of this work the authors used Copilot in order to organize content and improve language and readability. After using this tool/service, the authors reviewed and edited the content as needed and take full responsibility for the content of the published article.

## Credit authorship contribution statement

**Mary-Theresa Usuanlele:** Writing – original draft, Project administration, Methodology, Investigation, Formal analysis, Data curation, Conceptualization. **Conrad Kabali:** Writing – review & editing, Investigation, Data curation. **Lawrence Mbuagbaw:** Writing – review & editing, Supervision, Investigation, Conceptualization. **Mark Loeb:** Writing – review & editing, Supervision, Conceptualization. **Dominik Mertz:** Writing – review & editing, Supervision, Resources, Project administration, Methodology, Conceptualization.

## Sources of funding

This research did not receive any specific grant from funding agencies in the public, commercial, or not-for-profit sectors.

## Declaration of competing interest

The authors declare that they have no known competing financial interests or personal relationships that could have appeared to influence the work reported in this paper.

## Appendix A Supplementary data

Supplementary material: Appendix 1. Detailed Search StrategySupplementary Appendix 1 provides the complete search strategy used for all databases, including search terms, Boolean operators, filters, and date limits. This allows full reproducibility of the literature search. Appendix 2. Forest PlotsSupplementary Appendix 2 contains the forest plots for all meta-analyses conducted in the study, includingForest plot of study‑level prevalence estimates (no Pooled Estimate). Forest plot showing proportion of C. difficile colonization among symptomatic and asymptomatic individuals (no pooled estimates)Forest plot showing proportion of C. difficile colonization in adult, paediatric and mixed populations (no pooled estimates).
