# Supplementary material for: Clostridioides difficile in West Africa: a systematic review of the limited evidence and key knowledge gaps
Source: Glob Epidemiol. 2026 Aug 18;12:100281. doi: 10.1016/j.gloepi.2026.100281 (PMC13524661; doi:10.1016/j.gloepi.2026.100281)
Supplement: Supplementary file 1 [file mmc1.docx]

**APPENDIX 1. Detailed search strategy.**

***Supplementary Appendix 1 provides the complete search strategy used for all databases, including search terms, Boolean operators, filters, and date limits. This allows full reproducibility of the literature search.***

**Databases**

- PubMed <https://pubmed.ncbi.nlm.nih.gov/>
- Embase <https://www.embase.com>
- EBM Reviews & Cochrane Library via OVID: <https://www.wolterskluwer.com/en/solutions/ovid/evidencebased-medicine-reviews-ebmr-904> (EBM Reviews - ACP Journal Club <1991 to August 2023>, Cochrane Central Register of Controlled Trials <August 2023>, Cochrane Database of Systematic Reviews <2005 to September 6, 2023>, Cochrane Clinical Answers <August 2023>, Cochrane Methodology Register <3rd Quarter 2012>, Health Technology Assessment <4th Quarter 2016>, NHS Economic Evaluation Database <1st Quarter 2016>)

**Regional Repositories**

- African Journal Online. <https://www.ajol.info/index.php/ajol>
- WHO. Africa Index Medicus. <https://indexmedicus.afro.who.int/>

**Gray Publications**

- MedRxiv & BioRxiv <https://www.medrxiv.org/> - <https://www.biorxiv.org/>
- Clinical trials registry <https://clinicaltrials.gov/>

**Search limits**: none

**Database retrieval**

| **Databases** | **05/08/2024** | **18/09/2024** |
| --- | --- | --- |
| PubMed | 639 | 643 |
| Embase | 328 | 330 |
| EBM Reviews- via OVID | - | 3 |
| Africa Index Medicus | 62 | 62 |
| African Journal Online | Manual Search |  |
| MedRxiv & BioRxiv | 0 | 0 |
| EEUU Clinical trials | 2 | 2 |
| **TOTAL** | 1031 | **1040** |

**.RIS Archives attached – 05AGO2024**

- Clostridium_Pubmed_639r_05AGO2024.ris (also in .nbib)
- Clostridium_Embase_328r_05AGO2024.ris
- Clostridium_AIM_62r_05AGO2024
- Clostridium_ClinicalTrials_2r_05AGO2024.ris

**PubMed search:**

|  | **Search lines** | **HITS** |
| --- | --- | --- |
| **#1** | **"Clostridioides Difficile"[MeSH Terms]** OR "clostridioides difficile"[Title/Abstract] OR "clostridioides infections"[Title/Abstract] OR "clostridioides perfringens"[Title/Abstract] OR **"Clostridium Infections"[MeSH Terms]** OR "Clostridium Infections"[Title/Abstract] OR "clostridium difficile"[Title/Abstract] OR "clostridium perfringens"[Title/Abstract] OR "clostridium sordellii"[Title/Abstract] OR **"enterocolitis, pseudomembranous"[MeSH Terms]** OR "pseudomembranous colitis"[Title/Abstract] OR "pseudomembranous enteritis"[Title/Abstract] OR "pseudomembranous enterocolitis"[Title/Abstract] OR "antibiotic-associated colitis"[Title/Abstract] OR "antibiotic-associated diarrhea"[Title/Abstract] | [49,767](https://pubmed.ncbi.nlm.nih.gov/?term=%22Clostridioides+Difficile%22%5BMeSH+Terms%5D+OR+%22clostridioides+difficile%22%5BTitle%2FAbstract%5D+OR+%22clostridioides+infections%22%5BTitle%2FAbstract%5D+OR+%22clostridioides+perfringens%22%5BTitle%2FAbstract%5D+OR+%22Clostridium+Infections%22%5BMeSH+Terms%5D+OR+%22Clostridium+Infections%22%5BTitle%2FAbstract%5D+OR+%22clostridium+difficile%22%5BTitle%2FAbstract%5D+OR+%22clostridium+perfringens%22%5BTitle%2FAbstract%5D+OR+%22clostridium+sordellii%22%5BTitle%2FAbstract%5D+OR+%22enterocolitis%2C+pseudomembranous%22%5BMeSH+Terms%5D+OR+%22pseudomembranous+colitis%22%5BTitle%2FAbstract%5D+OR+%22pseudomembranous+enteritis%22%5BTitle%2FAbstract%5D+OR+%22pseudomembranous+enterocolitis%22%5BTitle%2FAbstract%5D+OR+%22antibiotic-associated+colitis%22%5BTitle%2FAbstract%5D+OR+%22antibiotic-associated+diarrhea%22%5BTitle%2FAbstract%5D&sort=relevance&ac=no) |
| **#2** | **"Benin"[MeSH Terms] OR "Burkina Faso"[MeSH Terms] OR "Cabo Verde"[MeSH Terms] OR "Gambia"[MeSH Terms] OR "Ghana"[MeSH Terms] OR "Guinea"[MeSH Terms] OR "guinea bissau"[MeSH Terms] OR "Cote d'Ivoire"[MeSH Terms] OR "Liberia"[MeSH Terms] OR "Mali"[MeSH Terms] OR "Mauritania"[MeSH Terms] OR "Niger"[MeSH Terms] OR "Nigeria"[MeSH Terms] OR "Senegal"[MeSH Terms] OR "Sierra Leone"[MeSH Terms] OR "Togo"[MeSH Terms] OR "africa, western"[MeSH Terms]** OR "Benin"[Title/Abstract] OR "Burkina Faso"[Title/Abstract] OR "Cape Verde"[Title/Abstract] OR "Cabo Verde"[Title/Abstract] OR "Gambia"[Title/Abstract] OR "Ghana"[Title/Abstract] OR "Guinea"[Title/Abstract] OR "Ivory Coast"[Title/Abstract] OR "Cote d'Ivoire"[Title/Abstract] OR "Liberia"[Title/Abstract] OR "Mali"[Title/Abstract] OR "Mauritania"[Title/Abstract] OR "Niger"[Title/Abstract] OR "Nigeria"[Title/Abstract] OR "Senegal"[Title/Abstract] OR "Sierra Leone"[Title/Abstract] OR "Togo"[Title/Abstract] OR "West Africa"[Title/Abstract] OR "Western Africa"[Title/Abstract] | [235,700](https://pubmed.ncbi.nlm.nih.gov/?term=%22Benin%22%5BMeSH+Terms%5D+OR+%22Burkina+Faso%22%5BMeSH+Terms%5D+OR+%22Cabo+Verde%22%5BMeSH+Terms%5D+OR+%22Gambia%22%5BMeSH+Terms%5D+OR+%22Ghana%22%5BMeSH+Terms%5D+OR+%22Guinea%22%5BMeSH+Terms%5D+OR+%22guinea+bissau%22%5BMeSH+Terms%5D+OR+%22Cote+d%27Ivoire%22%5BMeSH+Terms%5D+OR+%22Liberia%22%5BMeSH+Terms%5D+OR+%22Mali%22%5BMeSH+Terms%5D+OR+%22Mauritania%22%5BMeSH+Terms%5D+OR+%22Niger%22%5BMeSH+Terms%5D+OR+%22Nigeria%22%5BMeSH+Terms%5D+OR+%22Senegal%22%5BMeSH+Terms%5D+OR+%22Sierra+Leone%22%5BMeSH+Terms%5D+OR+%22Togo%22%5BMeSH+Terms%5D+OR+%22africa%2C+western%22%5BMeSH+Terms%5D+OR+%22Benin%22%5BTitle%2FAbstract%5D+OR+%22Burkina+Faso%22%5BTitle%2FAbstract%5D+OR+%22Cape+Verde%22%5BTitle%2FAbstract%5D+OR+%22Cabo+Verde%22%5BTitle%2FAbstract%5D+OR+%22Gambia%22%5BTitle%2FAbstract%5D+OR+%22Ghana%22%5BTitle%2FAbstract%5D+OR+%22Guinea%22%5BTitle%2FAbstract%5D+OR+%22Ivory+Coast%22%5BTitle%2FAbstract%5D+OR+%22Cote+d%27Ivoire%22%5BTitle%2FAbstract%5D+OR+%22Liberia%22%5BTitle%2FAbstract%5D+OR+%22Mali%22%5BTitle%2FAbstract%5D+OR+%22Mauritania%22%5BTitle%2FAbstract%5D+OR+%22Niger%22%5BTitle%2FAbstract%5D+OR+%22Nigeria%22%5BTitle%2FAbstract%5D+OR+%22Senegal%22%5BTitle%2FAbstract%5D+OR+%22Sierra+Leone%22%5BTitle%2FAbstract%5D+OR+%22Togo%22%5BTitle%2FAbstract%5D+OR+%22West+Africa%22%5BTitle%2FAbstract%5D+OR+%22Western+Africa%22%5BTitle%2FAbstract%5D&sort=relevance&ac=no) |
| **#3** | **#1 AND #2** | [**639**](https://pubmed.ncbi.nlm.nih.gov/?term=%28%22Clostridioides+Difficile%22%5BMeSH+Terms%5D+OR+%22Clostridioides+Difficile%22%5BTitle%2FAbstract%5D+OR+%22clostridioides+infections%22%5BTitle%2FAbstract%5D+OR+%22clostridioides+perfringens%22%5BTitle%2FAbstract%5D+OR+%22Clostridium+Infections%22%5BMeSH+Terms%5D+OR+%22Clostridium+Infections%22%5BTitle%2FAbstract%5D+OR+%22clostridium+difficile%22%5BTitle%2FAbstract%5D+OR+%22clostridium+perfringens%22%5BTitle%2FAbstract%5D+OR+%22clostridium+sordellii%22%5BTitle%2FAbstract%5D+OR+%22enterocolitis%2C+pseudomembranous%22%5BMeSH+Terms%5D+OR+%22pseudomembranous+colitis%22%5BTitle%2FAbstract%5D+OR+%22pseudomembranous+enteritis%22%5BTitle%2FAbstract%5D+OR+%22pseudomembranous+enterocolitis%22%5BTitle%2FAbstract%5D+OR+%22antibiotic-associated+colitis%22%5BTitle%2FAbstract%5D+OR+%22antibiotic-associated+diarrhea%22%5BTitle%2FAbstract%5D%29+AND+%28%22Benin%22%5BMeSH+Terms%5D+OR+%22Burkina+Faso%22%5BMeSH+Terms%5D+OR+%22Cabo+Verde%22%5BMeSH+Terms%5D+OR+%22Gambia%22%5BMeSH+Terms%5D+OR+%22Ghana%22%5BMeSH+Terms%5D+OR+%22Guinea%22%5BMeSH+Terms%5D+OR+%22guinea+bissau%22%5BMeSH+Terms%5D+OR+%22Cote+d%27Ivoire%22%5BMeSH+Terms%5D+OR+%22Liberia%22%5BMeSH+Terms%5D+OR+%22Mali%22%5BMeSH+Terms%5D+OR+%22Mauritania%22%5BMeSH+Terms%5D+OR+%22Niger%22%5BMeSH+Terms%5D+OR+%22Nigeria%22%5BMeSH+Terms%5D+OR+%22Senegal%22%5BMeSH+Terms%5D+OR+%22Sierra+Leone%22%5BMeSH+Terms%5D+OR+%22Togo%22%5BMeSH+Terms%5D+OR+%22africa%2C+western%22%5BMeSH+Terms%5D+OR+%22Benin%22%5BTitle%2FAbstract%5D+OR+%22Burkina+Faso%22%5BTitle%2FAbstract%5D+OR+%22Cape+Verde%22%5BTitle%2FAbstract%5D+OR+%22Cabo+Verde%22%5BTitle%2FAbstract%5D+OR+%22Gambia%22%5BTitle%2FAbstract%5D+OR+%22Ghana%22%5BTitle%2FAbstract%5D+OR+%22Guinea%22%5BTitle%2FAbstract%5D+OR+%22Ivory+Coast%22%5BTitle%2FAbstract%5D+OR+%22Cote+d%27Ivoire%22%5BTitle%2FAbstract%5D+OR+%22Liberia%22%5BTitle%2FAbstract%5D+OR+%22Mali%22%5BTitle%2FAbstract%5D+OR+%22Mauritania%22%5BTitle%2FAbstract%5D+OR+%22Niger%22%5BTitle%2FAbstract%5D+OR+%22Nigeria%22%5BTitle%2FAbstract%5D+OR+%22Senegal%22%5BTitle%2FAbstract%5D+OR+%22Sier) |
| **#4** | **"prevalence"[MeSH Terms]** OR "prevalence"[Title/Abstract] OR **"incidence"[MeSH Terms]** OR "incidence"[Title/Abstract] OR "rate*"[Title/Abstract] OR "occur*"[Title/Abstract] OR "frequenc*"[Title/Abstract] OR "proport*"[Title/Abstract] OR "number*"[Title/Abstract] OR "percent*"[Title/Abstract] | [10,276,395](https://pubmed.ncbi.nlm.nih.gov/?term=%22prevalence%22%5BMeSH+Terms%5D+OR+%22prevalence%22%5BTitle%2FAbstract%5D+OR+%22incidence%22%5BMeSH+Terms%5D+OR+%22incidence%22%5BTitle%2FAbstract%5D+OR+%22rate%2A%22%5BTitle%2FAbstract%5D+OR+occur%2A%5Btiab%5D+OR+frequenc%2A%5Btiab%5D+OR+proport%2A%5Btiab%5D+OR+number%2A%5Btiab%5D+OR+percent%2A%5Btiab%5D&sort=relevance&ac=no) |
| **#5** | **#3 AND #4** | [**225**](https://pubmed.ncbi.nlm.nih.gov/?term=longquery537d72deea10a40ed7ef&sort=relevance&ac=no) |
| #6 | #5 OR #3 | [639](https://pubmed.ncbi.nlm.nih.gov/?term=%236+OR+%234&sort=relevance&ac=no) |
|  | *All the 225 hits are included in the 639 exported*. 05AGO2024 |  |
|  | **Export 18SEP 2024** | [**643**](https://pubmed.ncbi.nlm.nih.gov/?term=%28%22Clostridioides+Difficile%22%5BMeSH+Terms%5D+OR+%22Clostridioides+Difficile%22%5BTitle%2FAbstract%5D+OR+%22clostridioides+infections%22%5BTitle%2FAbstract%5D+OR+%22clostridioides+perfringens%22%5BTitle%2FAbstract%5D+OR+%22Clostridium+Infections%22%5BMeSH+Terms%5D+OR+%22Clostridium+Infections%22%5BTitle%2FAbstract%5D+OR+%22clostridium+difficile%22%5BTitle%2FAbstract%5D+OR+%22clostridium+perfringens%22%5BTitle%2FAbstract%5D+OR+%22clostridium+sordellii%22%5BTitle%2FAbstract%5D+OR+%22enterocolitis%2C+pseudomembranous%22%5BMeSH+Terms%5D+OR+%22pseudomembranous+colitis%22%5BTitle%2FAbstract%5D+OR+%22pseudomembranous+enteritis%22%5BTitle%2FAbstract%5D+OR+%22pseudomembranous+enterocolitis%22%5BTitle%2FAbstract%5D+OR+%22antibiotic-associated+colitis%22%5BTitle%2FAbstract%5D+OR+%22antibiotic-associated+diarrhea%22%5BTitle%2FAbstract%5D%29+AND+%28%22Benin%22%5BMeSH+Terms%5D+OR+%22Burkina+Faso%22%5BMeSH+Terms%5D+OR+%22Cabo+Verde%22%5BMeSH+Terms%5D+OR+%22Gambia%22%5BMeSH+Terms%5D+OR+%22Ghana%22%5BMeSH+Terms%5D+OR+%22Guinea%22%5BMeSH+Terms%5D+OR+%22guinea+bissau%22%5BMeSH+Terms%5D+OR+%22Cote+d%27Ivoire%22%5BMeSH+Terms%5D+OR+%22Liberia%22%5BMeSH+Terms%5D+OR+%22Mali%22%5BMeSH+Terms%5D+OR+%22Mauritania%22%5BMeSH+Terms%5D+OR+%22Niger%22%5BMeSH+Terms%5D+OR+%22Nigeria%22%5BMeSH+Terms%5D+OR+%22Senegal%22%5BMeSH+Terms%5D+OR+%22Sierra+Leone%22%5BMeSH+Terms%5D+OR+%22Togo%22%5BMeSH+Terms%5D+OR+%22africa%2C+western%22%5BMeSH+Terms%5D+OR+%22Benin%22%5BTitle%2FAbstract%5D+OR+%22Burkina+Faso%22%5BTitle%2FAbstract%5D+OR+%22Cape+Verde%22%5BTitle%2FAbstract%5D+OR+%22Cabo+Verde%22%5BTitle%2FAbstract%5D+OR+%22Gambia%22%5BTitle%2FAbstract%5D+OR+%22Ghana%22%5BTitle%2FAbstract%5D+OR+%22Guinea%22%5BTitle%2FAbstract%5D+OR+%22Ivory+Coast%22%5BTitle%2FAbstract%5D+OR+%22Cote+d%27Ivoire%22%5BTitle%2FAbstract%5D+OR+%22Liberia%22%5BTitle%2FAbstract%5D+OR+%22Mali%22%5BTitle%2FAbstract%5D+OR+%22Mauritania%22%5BTitle%2FAbstract%5D+OR+%22Niger%22%5BTitle%2FAbstract%5D+OR+%22Nigeria%22%5BTitle%2FAbstract%5D+OR+%22Senegal%22%5BTitle%2FAbstract%5D+OR+%22Sier) |

**Embase search:**

|  | **Search lines** | **HITS** |
| --- | --- | --- |
| #1 | 'benin'/exp OR 'benin' OR 'burkina faso'/exp OR 'burkina faso' OR 'cabo verde'/exp OR 'cabo verde' OR 'gambia'/exp OR 'gambia' OR 'ghana'/exp OR 'ghana' OR 'guinea'/exp OR 'guinea' OR 'guinea bissau'/exp OR 'guinea bissau' OR 'cote de ivoire' OR 'liberia'/exp OR 'liberia' OR 'mali'/exp OR 'mali' OR 'mauritania'/exp OR 'mauritania' OR 'niger'/exp OR 'niger' OR 'nigeria'/exp OR 'nigeria' OR 'senegal'/exp OR 'senegal' OR 'sierra leone'/exp OR 'sierra leone' OR 'togo'/exp OR 'togo' OR 'west africa'/exp OR 'west africa' | [432,150](https://www-embase-com.ezproxy.unal.edu.co/) |
| #2 | 'clostridioides difficile'/exp OR 'clostridioides difficile' OR 'clostridium infection'/exp OR 'clostridium infection' OR 'pseudomembranous colitis'/exp OR 'pseudomembranous colitis' OR 'clostridioides perfringens' OR 'clostridium perfringens'/exp OR 'clostridium perfringens' OR 'clostridium perfringens infection'/exp OR 'clostridium perfringens infection' OR 'clostridium sordellii'/exp OR 'clostridium sordellii' OR 'pseudomembranous colitis'/exp OR 'pseudomembranous colitis' OR 'pseudomembranous enteritis' OR 'antibiotic-associated colitis' OR 'antibiotic associated diarrhea'/exp OR 'antibiotic associated diarrhea' | [83,732](https://www-embase-com.ezproxy.unal.edu.co/) |
| #3 | 'prevalence'/exp OR 'prevalence' OR 'incidence'/exp OR 'incidence' OR 'occurrence' OR 'frequency'/exp OR 'frequency' OR 'proportion' OR 'number'/exp OR 'number' OR 'percentage' | [8,248,785](https://www-embase-com.ezproxy.unal.edu.co/) |
| #4 | (#1 AND #2) OR (#1 AND #2 AND #3)  AND [embase]/lim NOT ([embase]/lim AND [medline]/lim)  *Export*. 05AGO2024 | [328](https://www-embase-com.ezproxy.unal.edu.co/) |
|  | **Export 18SEP 2024** | **330** |

**EBM Reviews via OVID search:**

|  | **Search lines** | **HITS** |
| --- | --- | --- |
| #1 | (Benin or Burkina Faso or Cabo Verde or Gambia or Ghana or Guinea or "Ivory Coast" or Liberia or Mali or Mauritania or Niger or Nigeria or Senegal or "Sierra Leone" or Togo or "West Africa" or "Western Africa").ab. | **5317** |
| #2 | "Clostridioides Difficile".ab. or "Clostridioides Difficile".hw. or "Clostridioides Difficile".ti. or "clostridioides infections".ab. or "clostridioides infections".hw. or "clostridioides infections".ti. or "clostridioides perfringens".ab. or "clostridioides perfringens".hw. or "clostridioides perfringens".ti. or "Clostridium Infections".ab. or "Clostridium Infections".hw. or "Clostridium Infections".ti. or "clostridium difficile".ab. or "clostridium difficile".hw. or "clostridium difficile".ti. or "pseudomembranous colitis".ab. or "pseudomembranous colitis".hw. or "pseudomembranous enteritis".ab. or "pseudomembranous enteritis".hw. or "pseudomembranous enterocolitis".ab. or "pseudomembranous enterocolitis".hw. or "antibiotic-associated colitis".ab. or "antibiotic-associated colitis".hw. or "antibiotic-associated diarrhea".ab. or "antibiotic-associated diarrhea".hw. | **1993** |
| **#3** | **#1 AND #2**  EBM Reviews - ACP Journal Club <1991 to August 2024>  **EBM Reviews - Cochrane Central Register of Controlled Trials <August 2024>**  EBM Reviews - Cochrane Database of Systematic Reviews <2005 to September 18, 2024>  EBM Reviews - Cochrane Clinical Answers <August 2024>  EBM Reviews - Cochrane Methodology Register <3rd Quarter 2012> | **3** |
|  | **Export 18 SEP 2024** | **3** |

**African Index Medicus:**

|  | **Search lines** | **HITS** |
| --- | --- | --- |
| #1 | tw:((clostridioides difficile) OR (clostridium infection) OR (pseudomembranous colitis) OR (clostridioides perfringens) OR (clostridium perfringens) OR (clostridium sordellii) OR (pseudomembranous colitis) OR (pseudomembranous enteritis) OR (antibiotic-associated colitis) OR (antibiotic associated diarrhea)) | **62** |
| ***#1*** | **Without limits or geographic line**  *Export*. 05AGO2024 | **62** |

**MedRxiv and BioRxiv search:**

|  | "clostridioides difficile" OR "clostridium infection" OR "pseudomembranous enterocolitis" | **0** |
| --- | --- | --- |
| **TOTAL** | All the main conditions terms were searched in both repositories with and without geographic line but there are no hits. 05AGO2024 | **0** |

**EEUU Clinical Trials search:**

|  | (Clostridioides Difficile OR Clostridium Infections) in Mali  (Clostridioides Difficile OR Clostridium Infections) in Guinea-Bissau | **1**  **1** |
| --- | --- | --- |
| **TOTAL** | All the countries and Africa terms were combined with the main conditions terms, but only these countries have hits. Export 05AGO2024 | **2** |

**African Journals Online**

The portal does not allow export the results. **Therefore, you will have to do the selection directly on the website.** The search in the portal is powered by Google, so there is no possibility to do the search lines combinations used in the other databases.

**Every link, shows the hits from every search term used.**

**clostridioides difficile – 15 HITS**

[**https://www.ajol.info/index.php/ajol/Gsearch/google?q=clostridioides+difficile+infection#gsc.tab=0&gsc.q=clostridioides%20difficile&gsc.sort=&gsc.page=1**](https://www.ajol.info/index.php/ajol/Gsearch/google?q=clostridioides+difficile+infection#gsc.tab=0&gsc.q=clostridioides%20difficile&gsc.sort=&gsc.page=1)

**clostridium infections humans – 244 HITS**

[**https://www.ajol.info/index.php/ajol/Gsearch/google?q=clostridioides+difficile+infection#gsc.tab=0&gsc.q=clostridium%20infections%20humans&gsc.sort=**](https://www.ajol.info/index.php/ajol/Gsearch/google?q=clostridioides+difficile+infection#gsc.tab=0&gsc.q=clostridium%20infections%20humans&gsc.sort=)

**clostridium sordellii infections – 3HITS**

[**https://www.ajol.info/index.php/ajol/Gsearch/google?q=clostridioides+difficile+infection#gsc.tab=0&gsc.q=clostridium%20sordellii%20infections&gsc.sort=**](https://www.ajol.info/index.php/ajol/Gsearch/google?q=clostridioides+difficile+infection#gsc.tab=0&gsc.q=clostridium%20sordellii%20infections&gsc.sort=)

**clostridium perfringens infections – 222HITS**

[**https://www.ajol.info/index.php/ajol/Gsearch/google?q=clostridioides+difficile+infection#gsc.tab=0&gsc.q=clostridium%20perfringens%20infections&gsc.sort=**](https://www.ajol.info/index.php/ajol/Gsearch/google?q=clostridioides+difficile+infection#gsc.tab=0&gsc.q=clostridium%20perfringens%20infections&gsc.sort=)

**clostridium difficile in humans – 643 HITS**

[**https://www.ajol.info/index.php/ajol/Gsearch/google?q=clostridioides+difficile+infection#gsc.tab=0&gsc.q=clostridium%20difficile%20in%20humans&gsc.sort=**](https://www.ajol.info/index.php/ajol/Gsearch/google?q=clostridioides+difficile+infection#gsc.tab=0&gsc.q=clostridium%20difficile%20in%20humans&gsc.sort=)

**APPENDIX 2. FOREST PLOTS**

***Supplementary Appendix 2 contains the forest plots for all meta-analyses conducted in the study, including***

a) ***Forest plot of study‑level prevalence estimates (no Pooled Estimate).***
b) ***Forest plot showing  proportion of C. difficile colonization among symptomatic and asymptomatic individuals (no pooled estimates)***
c) ***Forest plot showing proportion of C. difficile colonization in adult, paediatric and mixed populations (no pooled estimates).***

Fig 2: Forest plot of study‑level prevalence estimates (no Pooled Estimate).

Fig 3: Forest plot showing proportion of *C. difficile* colonization among symptomatic and asymptomatic individuals (no pooled estimates)

Fig 4: Forest plot showing proportion of *C. difficile* colonization in adult, paediatric and mixed populations (no pooled estimates).
